# A continuous-flow protocol for photoredox-catalyzed multicomponent Petasis reaction

**DOI:** 10.1016/j.xpro.2022.101162

**Published:** 2022-02-05

**Authors:** Monica Oliva, Frederick Martens, Erik V. Van der Eycken, Upendra Kumar Sharma

**Affiliations:** 1 Laboratory for Organic & Microwave-Assisted Chemistry (LOMAC), Department of Chemistry, KU Leuven, Celestijnenlaan 200F, 3001 Leuven, Belgium; 2 Peoples’ Friendship University of Russia (RUDN University), Miklukho-Maklaya street 6, Moscow 117198, Russia

**Keywords:** Chemistry, Environmental sciences

## Abstract

Here, we present a robust protocol for the facile and rapid synthesis of functionalized secondary amines in continuous flow. More specifically, we describe a detailed guide to perform a photocatalyzed Petasis reaction within 50 min using alkyl boronic acid as radical precursor and a Vapourtec E-series as key equipment. The desired functionalized amine has been synthesized in mmol scale and with a productivity rate of 0.2 mmol/h. The protocol is limited to alkyl boronic acids.

For complete details on the generation and use of this protocol, please refer to Oliva et al. (2021).

## Highlights

• Photoredox-catalyzed multicomponent Petasis reaction in continuous flow
• Synthesis of functionalized secondary amines in mmol scale
• Successful application of alkyl boronic acids as radical precursors in flow
• Protocol is limited to alkyl boronic acids

## Graphical abstract

**Figure:**
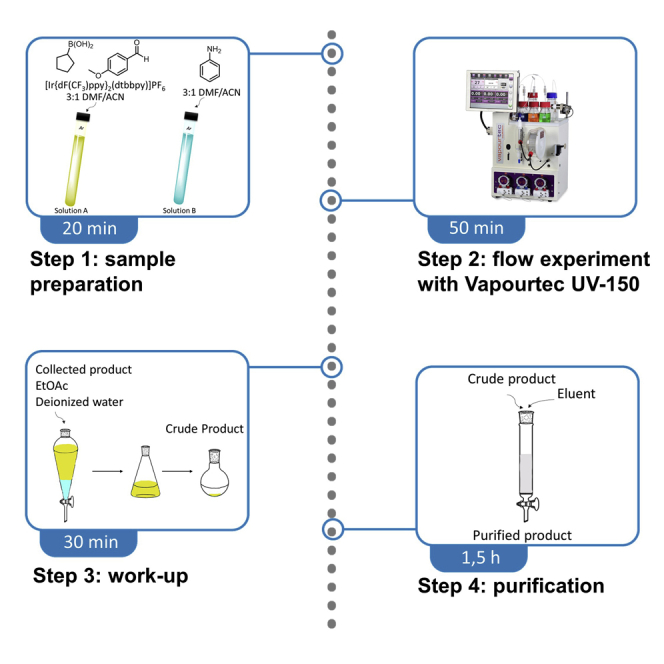

## Before you begin

Discovered in 1993 by Petasis and Akritopoulou, the Petasis reaction, also called boron-Mannich reaction, is a three-component reaction of a secondary amine, paraformaldehyde, and a vinyl- or phenyl boronic acid to form functionalized amines *via* a nucleophilic boron “ate” complex (Petasis and Akritopoulou, 1993). This method has been widely exploited in the last three decades (Wu et al., 2019), however, the inherent requirement of a directing group to form the borate intermediate, and the necessity to stabilize the negative charge on the migrating group severely restrict the broad utilization of this reaction.

In 2019, Molander and co-workers showed for the first time a photoredox catalyzed Petasis reaction, coupling anilines and benzaldehyde derivatives with alkyl trifluoroborate salts as radical precursors (Yi et al., 2019). With this new strategy in hand, they eliminated the need for a directing group installed on the boronic component, further giving access to milder reaction conditions and broader scope.

Recently, our group contributed to this field by applying alkyl boronic acids as efficient radical precursors as well as optimal substrates for application in flow, due to their inherent solubility in most organic solvents (Oliva et al., 2021). It is worth mentioning that the use of free boronic acids as an alkyl radical source has been overlooked due to their high oxidation potential (Li et al., 2016). Nonetheless, our group has recently reported the generation of alkyl radicals from free boronic acids by modulating their oxidation potential through hydrogen bonding interactions with an amide-based solvent (Ranjan et al., 2021).

In the last few decades, a constant positive evaluation of the merits of continuous-flow chemistry compared to batch has led to a significant increase in synthetic strategies and reactions developed in microreactors (Hartman et al., 2011; Baumann et al., 2020; De Risi et al., 2020; Hu, 2021). One of the benefits of continuous-flow technology is the possibility to run reactions in micro-diameter reactors, with consequent better heat and mass transport phenomena which can be translated into faster reaction times (Plutschack et al., 2017). The high surface area to volume ratio enables almost instantaneous heating or cooling and therefore better temperature control.

Therefore, the current protocol describes the specific steps for the synthesis of *N*-(cyclopentyl(4-methoxyphenyl)methyl)aniline in mmol scale, our selected model substrate for batch optimization, through a photocatalyzed multicomponent Petasis reaction using alkyl boronic acid as radical precursor and a Vapourtec E-series as flow equipment. The protocol has been extended to the synthesis of three additional substrates. For the same experiment performed in batch, please refer to Oliva et al. (2021).

### Preparation of the reagents and equipment

A complete list of reagents and equipment can be found in the “key resources table” and “materials and equipment”.

### Preparation of the reagent reservoirs

**Timing: 20 min**

In this step, two reagent reservoirs for the reaction are prepared. One screw cap test tube labeled as “reagent A” containing a solution of cyclopentyl boronic acid, the photocatalyst and *p*-anisaldehyde, and a second screw cap test tube labeled as “reagent B” containing a solution of aniline.

***Note:*** reagent reservoirs need to be made fresh every time.

1. Preparation of reagent A (Table 1)
  a. In one 10 mL screw cap test tube weight out 68 mg (0.6 mmol) of cyclopentyl boronic acid.
  b. Add 8 mg of [Ir{dF(CF_3_)ppy}_2_(dtbbpy)]PF_6_ (0.007 mmol).
  c. Seal the tube with a cap containing a PTFE-lined silicone septum. Place the reaction mixture under vacuum *via* an inlet needle connected to an argon/vacuum Schlenk manifold. Refill the test tube with Argon.
    ***Note:*** Nitrogen can be used as well to place the reaction under inert atmosphere.
  d. Repeat the evacuation-refill cycle three times, so that the reaction mixture is under Argon atmosphere.
  e. Add to the test tube 2 mL of a mixture 3:1 of dry *N*,*N*-dimethylformamide and dry acetonitrile with a syringe.
  f. Add 24 μL (0.2 mmol) of *p*-anisaldehyde with a microsyringe.
    **CRITICAL:** All solid reactants need to be completely solubilized in the reaction solvent, to avoid clogging issues.

**Table 1:** Preparation of reagent A solution

Chemical	Amount
Cyclopentyl boronic acid	68 mg
[Ir{dF(CF_3_)ppy}_2_(dtbbpy)]PF_6_	8 mg
*p*-anisaldehyde	24 μL
3:1 mixture *N*,*N*-dimethylformamide and acetonitrile	2 mL

2. Preparation of reagent B (Table 2)
  a. In a 10 mL screw cap test tube add with a Hamilton syringe 27 μL of aniline (0.3 mmol).
  b. Add to the test tube 2 mL of a mixture 3:1 of dry *N*,*N*-dimethylformamide and dry acetonitrile with a syringe.
  c. Seal the tube with a cap containing a PTFE-lined silicone septum. Place the reagent reservoir under argon *via* an inlet needle. Add an outlet needle and let degas the mixture for 5 min.

**Table 2:** Preparation of reagent B solution

Chemical	Amount
Aniline	27 μL
3:1 mixture *N*,*N*-dimethylformamide and acetonitrile	2 mL

***Note:*** Photoredox catalyzed reactions usually require inert atmosphere to avoid the deactivation of the photocatalyst’s triplet excited state by oxygen. Anaerobic conditions can be achieved by degassing the solvents prior addition to the sealed test tube. Nevertheless, in the present protocol, solvents were not degassed: controls experiments showed no influence on the outcome of the performed reaction.

### Vapourtec E-series set-up

**Timing: 10 min**

In this step, besides setting up the flow reactor to perform the reaction under optimal conditions, some key parameters such as pump speed, temperature and pressure are also set.

**Figure 1:**
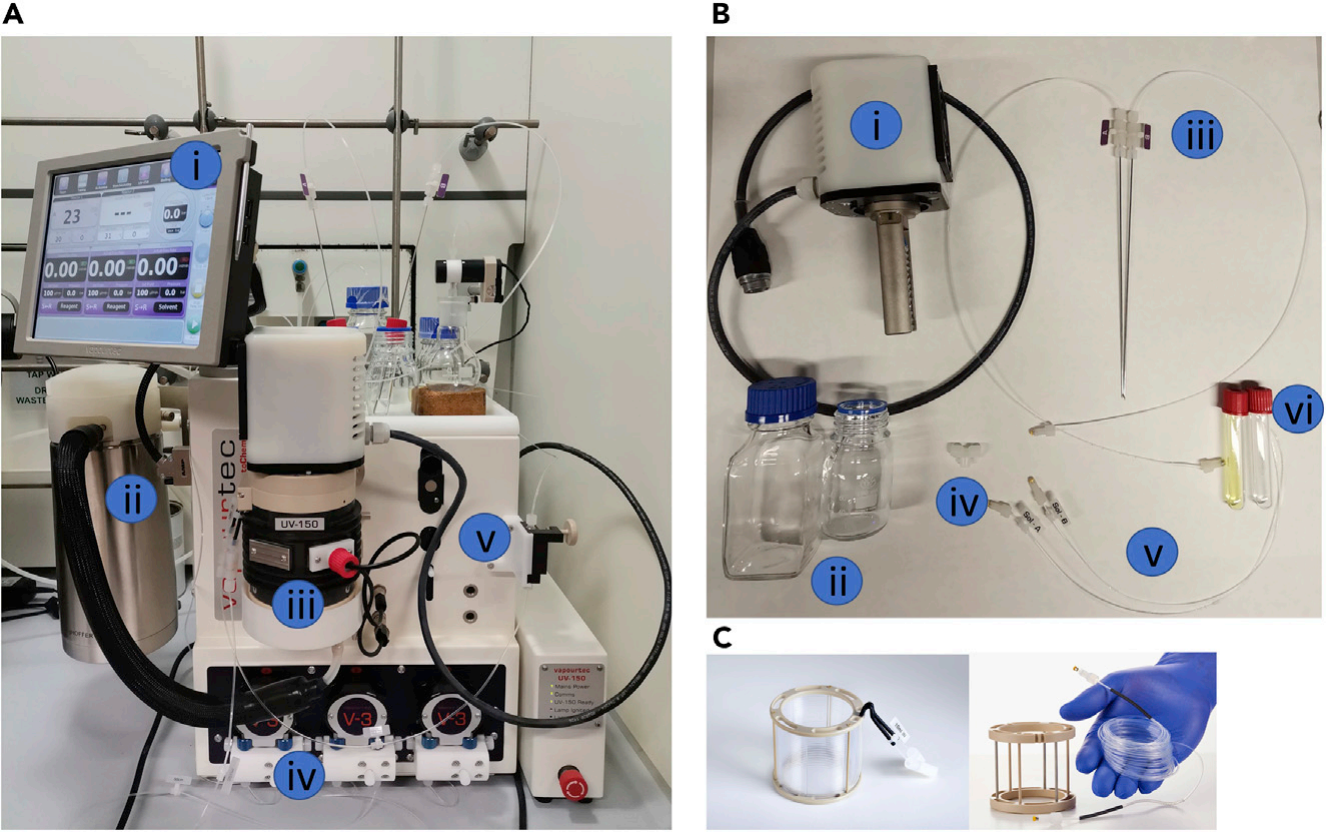
Overview of the flow system (A) Vapourtec E-series system: (i) touch screen interface, (ii) cooling module, (iii) UV-150 LED reactor, (iv) V-3 pumps, (v) back pressure regulator. (B) Zoom in on the required accessories: (i) high-power blue lamp, (ii) solvent and waste bottles, (iii) needles for the reagents reservoirs, (iv) T-mixer, (v) solvent lines, (vi) reagents reservoirs. (c) Zoom in on the reactor coil placed inside the UV-150 LED reactor.
  b. Select two blue tubes for the pumps to perform this reaction.
    **Pause point:** Vapourtec E-series pumps can be equipped with two different types of tubes, referred to as Red and Blue, compatible with different solvents. Blue tubes are suitable to pump *N,N*-dimethylformamide solvent based reaction mixtures.
  c. Fully open the back-pressure regulator.
  d. Place at the end point of the “waste” line and “collecting” line respectively, a 500 mL and 100 mL flask.
  e. Check on the touch screen interface that the exit stream is set to go to “waste”.
  f. Prepare 250 mL of a mixture 3:1 of dry *N*,*N*-dimethylformamide and dry acetonitrile and pour it in a 500 mL flask labeled as “Solvent”.
  g. Make sure that no air is in the system by priming the solvent, reagents tubes and the reactor.

3. Set-up the flow unit
  a. Equip the E-series with a 10-mL capacity PFA reactor coil (internal diameter = 0.05 inches) and a high-power 450 nm LED light source (Figure 1).

4. The cooling module
  a. Fill the cooling module with dry ice. A full tank would require approximately 2 kg of dry ice.
  b. Ensure the gas valve is turned on.
  c. Let flow the incoming N_2_ gas supply at a minimum pressure of 2 bar.

***Note:*** The cooling function is provided by flowing dry nitrogen through a bed of dry ice at low pressure. The mixture of dry nitrogen and carbon dioxide cools down around −78°C and it is fed intermittently through a pinch valve into the cooled reactor assembly. Through this assembly, the cold gas can be recirculated around the reactor coil to provide heat transfer between the gas and the reactor.

**Figure 2:**
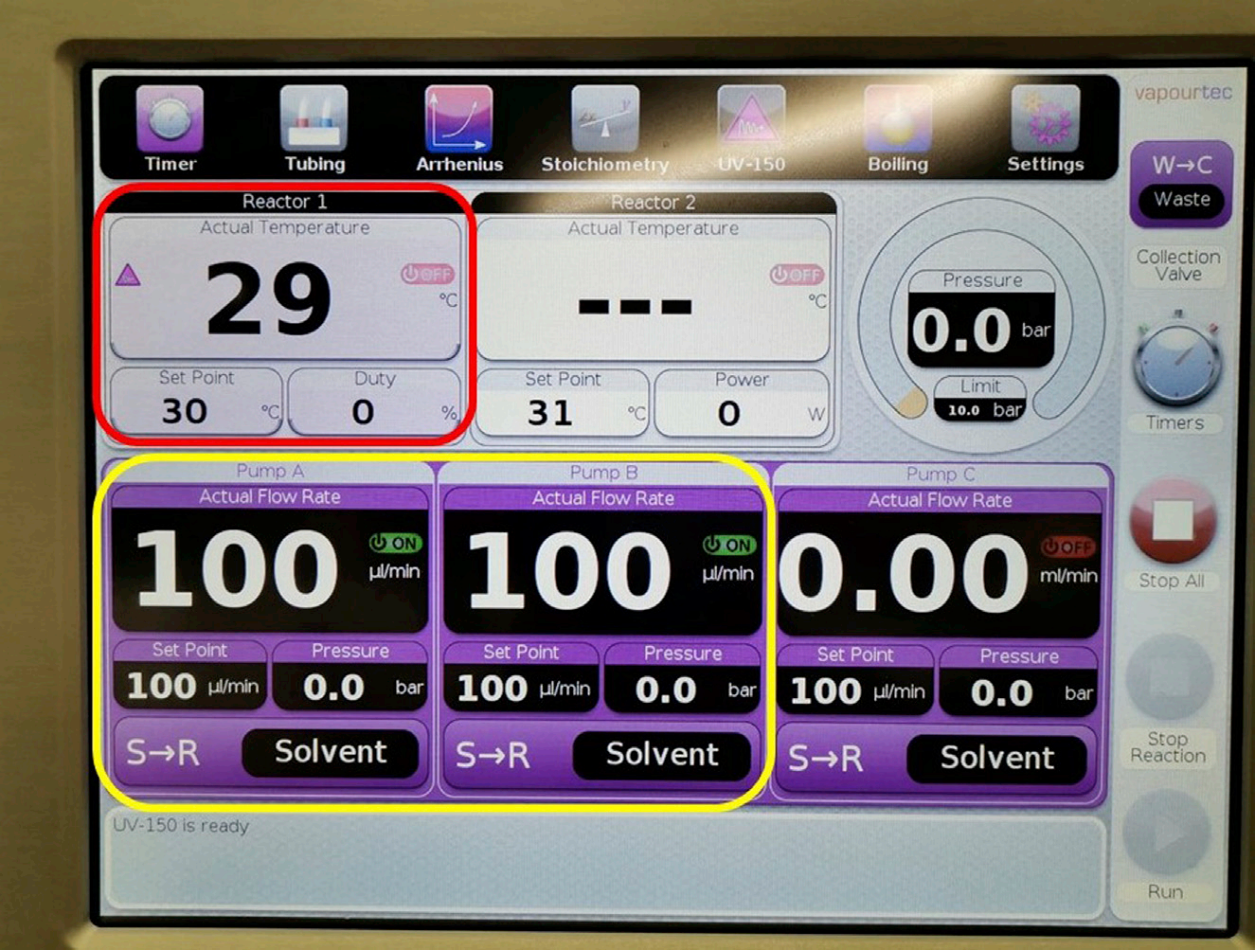
Vapourtec touch screen interface. Red box: reactor controls. Yellow box: pump A and B controls
  b. Set the flow rate of both pumps at 100 μL/min.
  c. Set the reactor temperature at 30°C (Figure 2, red box).
  d. Turn on the lamp.

5. Touch screen interface
  a. Turn on pump A and pump B (Figure 2, yellow box).

## Key resources table

**Table:** 

REAGENT or RESOURCE	SOURCE	IDENTIFIER
**Chemicals, peptides, and recombinant proteins**		

Cyclopentylboronic acid	Fluorochem	Cat#011025
*p*-Anisaldehyde	ACROS ORGANICS	Cat#10010680
Aniline	ACROS ORGANICS	Cat#10667512
*N,N*-Dimethylformamide	ACROS ORGANICS	Cat#10534341
Acetonitrile	ACROS ORGANICS	Cat#10222052
[Ir{dF(CF_3_)ppy}_2_(dtbbpy)]PF_6_	Synthesized in our lab	(Kelly et al., 2017) https://doi.org/10.1038/nprot.2016.176

**Other**		

Vapourtec easy-Photochem LED equipped with 2 peristaltic pumps	Vapourtec	Cat#50-1319
Blue V3 pump tubes	Vapourtec	Cat#50-1301
Vapourtec UV-150 LED-10 mL PFA reactor coil	Vapourtec	Cat#50-1580
450 nm LED light source. Input power 150 watt, radiant power 108 watt	Vapourtec	Cat#50-0299
High power LED power supply	Vapourtec	Cat#50-0295
Vapourtec E-series cooling module	Vapourtec	Cat#50-1314
E-series septum piercing kit	Vapourtec	Cat#50-1333
Septa 16,8mm Sil/PTFE 3,2mm for GL18	Fisher scientific	Cat#11834990
25 μL microliter syringe	Hamilton	Cat#80465
Argon/vacuum Schlenk manifold	Glasatelier Saillart	Cat#AN51211415
Thin layer chromatography using TLC-Plates ALUGRAM Xtra SIL G/UV254	MACHEREY-NAGEL	Cat#818333
Silica gel for chromatography, 0.060–0.200 mm, 60A	ACROS Organics	Cat#240370300
AV-III HD 400 (400 MHZ) spectrometer	Bruker	N/A

## Materials and equipment

### Reagents

• Cyclopentylboronic acid
• *p*-Anisaldehyde
• Aniline
• [Ir{dF(CF_3_)ppy}_2_(dtbbpy)]PF_6_
• Anhydrous *N,N*-Dimethylformamide
• Anhydrous Acetonitrile

### Equipment

• Vapourtec easy-Photochem LED equipped with 2 peristaltic pumps.
• Two blue V3 pump tubes.
• Vapourtec UV-150 LED-10 mL photochemical coil reactor.
• 450 nm LED light source. Input power 150 watt, radiant power 108 watt.
• High power LED power supply.
• Vapourtec E-series cooling module.
• E-series septum piercing kit.
• Reagents and solvent bottles.
• Round bottom flasks.
• Argon/vacuum Schlenk manifold.
• Microsyringes.
• Dry ice.

## Step-by-step method details

### Part 1: Synthesis of N-(cyclopentyl(4-methoxyphenyl)methyl)aniline

**Timing: 50 min**

**Scheme 1:**
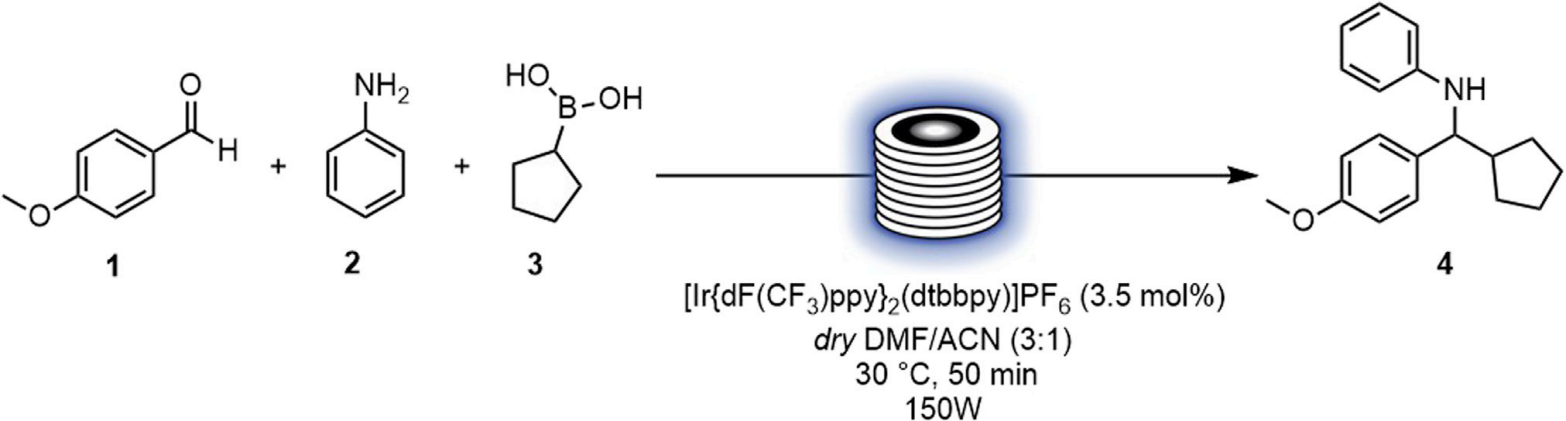
General scheme of the reaction (Oliva et al., 2021)

In this step, the synthesis of *N*-(cyclopentyl(4-methoxyphenyl)methyl)aniline **4** (Scheme 1) has been accomplished within 50 min under blue-light irradiation in flow.

1. Priming the pumps Troubleshooting 1
  a. Place “solvent A”, “reagent A”, “solvent B” and “reagent B” lines into the flask labeled “Solvent”.
  b. Turn on the flow unit and prime reagents and solvent lines.

**Figure 3:**
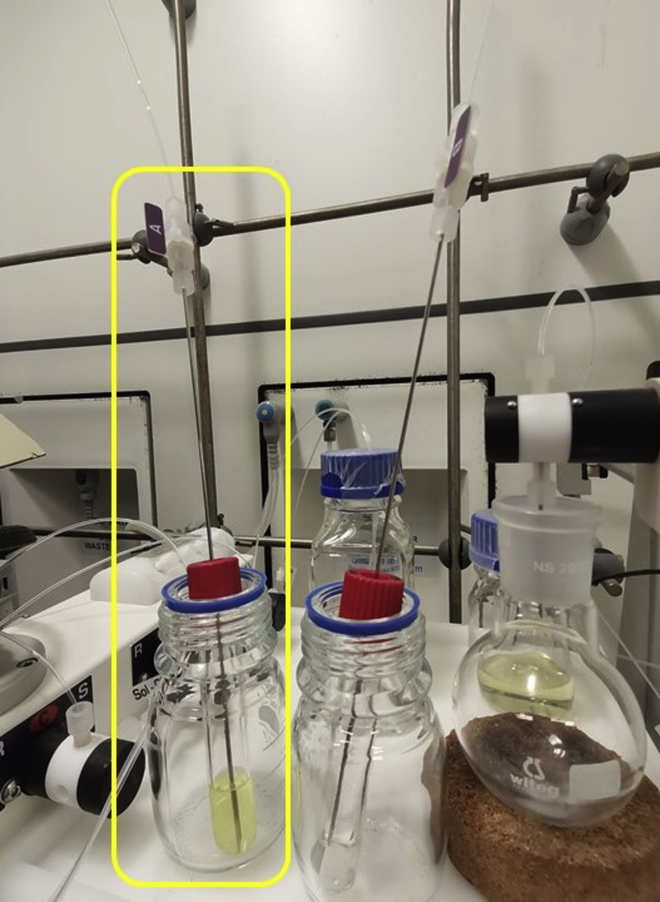
Needles inserted in the vial reaction

***Note:*** Connect to the end of the reagent lines a needle (Figure 3).

**Figure 4:**
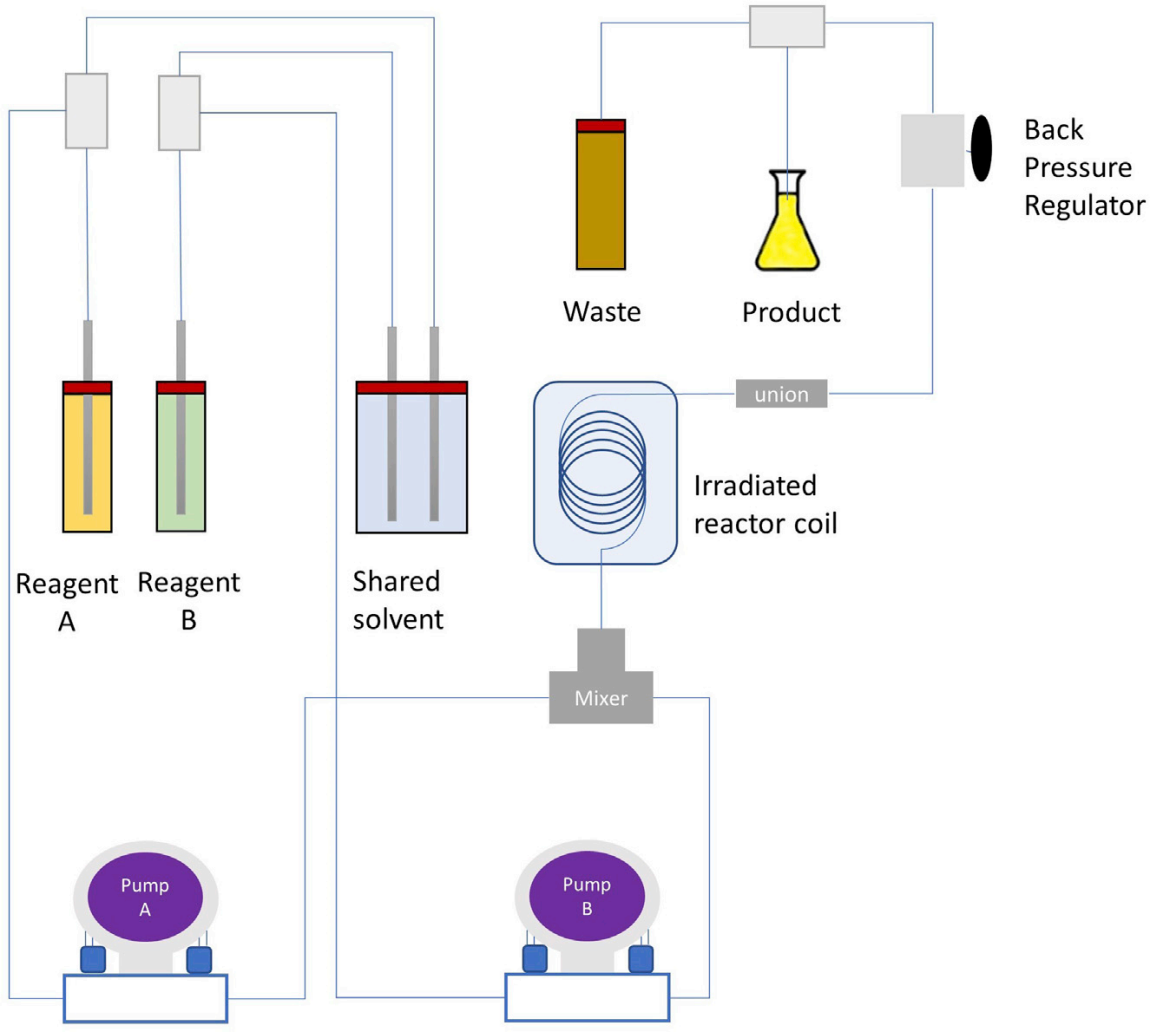
Schematic representation of the reaction in flow

2. Set up the reaction (Figure 4)
  a. Move one reagent line from the solvent flask to solution A.
  b. Move the second reagent line from the solvent flask to solution B.
  c. Ensure that both pumps are set to “solvent” on the screen.
  d. Press the button “Start” on the touch screen.

**Pause point:** Once the flow equipment reaches the desired temperature and the pressure is stable, the unit is ready to run the reaction.

3. Run the reaction Troubleshooting 2, 3, and 4
  a. Switch each of the pumps from “solvent” to “reagent”.
    **CRITICAL:** The time needed to start collecting the reaction mixture must be calculated in case the flow unit is not equipped with an automatic program.
  b. After 20 min, switch each pump from “reagent” to “solvent”.
  c. At the calculated time, switch the product stream from “waste” to “collect”.
  d. Collect for 50 additional minutes after loading of reagents is completed (step 3b).
  e. Switch from “collect” to “waste” after the whole reaction mixture is calculated to have gone through the system.
  f. Clean the system by continuing flushing solvent.

### Part 2: Purification of the crude material

**Timing: 2 h**

4. Upon reaction completion (approximately 1 h), transfer the reaction to a 250 mL separatory funnel and add 40 mL of deionized water and 40 mL of EtOAc. Shake the separatory funnel vigorously and let separate the aqueous phase from the organic one.
5. Transfer the organic phase and the aqueous phase in two separate 200 mL Erlenmeyer flasks.
6. Place the aqueous phase back to the separatory funnel and add to it 20 mL of EtOAc.
7. Shake vigorously the separatory funnel, and once the two phases are separated, transfer them in their respective Erlenmeyer flasks.
8. Repeat steps 6 and 7 two times.
9. Transfer the organic phase in the separatory funnel and add to it 40 mL of deionized water. Shake the separatory funnel vigorously and let separate the aqueous phase from the organic one.
10. Discard the aqueous phase in the appropriate waste container.
11. Repeat steps 9 and 10 twice.
12. Transfer the organic phase in the separatory funnel, add to it 40 mL of Brine and repeat step 11.
13. Transfer the organic layer in an Erlenmeyer flask, add NaHSO_4_, gently shake the solution and filter it in a 100 mL round bottom flask.
14. Remove the solvent by rotatory evaporation (44°C, 235 mmHg, ∼15 min).
15. Add to the dried crude 1 mL of dichloromethane and 200 mg of silica. Gently swirl the flask and evaporate the solvent under vacuum (38°C, 800 mmHg, ∼10 min).
16. Purify the crude product by flash column chromatography (12 cm of silica, Ø of the column= 2.5 cm) using 98:2 (by volume) mixture of heptane/ethyl acetate (∼200 mL).
17. Collect the combined fractions containing pure product and concentrate under reduced pressure to yield the desired product. Troubleshooting 5

## Expected outcomes

*N*-(cyclopentyl(4-methoxyphenyl)methyl)aniline **4** appears as a yellow oil obtained in 85% yield (throughput = 57 mg/h).

### Analytical data

^**1**^**H NMR** (400 MHz, Chloroform-d) δ 7.29–7.23 (m, 2H), 7.10–7.03 (m, 2H), 6.87–6.81 (m, 2H), 6.65–6.58 (m,1H), 6.54–6.47 (m, 2H), 4.15 (s, 1H), 4.04 (d, J = 8.4 Hz, 1H), 3.78 (s, 3H), 2.21–2.08 (m, 1H), 1.89 (dtd, J =11.9, 7.4, 3.4 Hz, 1H), 1.70–1.56 (m, 3H), 1.52–1.40 (m, 3H), 1.32–1.24 (m, 1H).

^**13**^**C NMR** (101 MHz, Chloroform-d) δ 158.52, 147.84, 136.10, 129.13, 128.02, 117.06, 113.81, 113.41, 62.59,55.32, 48.03, 30.23, 30.11, 25.39, 25.31.

**HRMS**(ESI^+^): [M-H] calculated for C_19_H_23_NO: 280.1707, found: 280.1702.

**IR** (neat, ν/cm^-1^) 3401, 2955, 2864, 1601, 1503, 1239, 1061, 749, 510.

## Limitations

The protocol is limited to alkyl boronic acids.

## Troubleshooting

### Problem 1

Step 1b: The pressure shown on the display is above the limit (10 bar).

### Potential solution

An inefficient cleaning of the tubing could cause the deposit of solid material on the inner surface of the reactor and consequent clogging with raising of the pressure. Always clean properly the tubes by flushing solvent at the end of every reaction. If this doesn’t solve the problem, check that the tubing connections to the back pressure regulator are tightened just enough to form a seal. If this still does not solve the issue, remove the back pressure regulator.

### Problem 2

Step 3a: The pumps can’t maintain a stable flow rate.

### Potential solution

Check that no air is inside the reactor module by priming the pumps with at least 10 mL of solvent. If the problem persists, recalibrate the pumps.

### Problem 3

Step 3b: The temperature can’t be maintained at the desired value but increases exponentially.

### Potential solution

Check that the nitrogen supply valve connected to the cooling module is open and set to 2 bar pressure. Check that the nozzle for cooled operation on the reactor module is correctly installed.

### Problem 4

Step 3a: Pumps are operational, but the needle inserted in the reaction vial can’t pump the mixture through the reactor.

### Potential solution

It is highly likely that the needle is stuck with solid particles from previous operations. Remove the needle and connect it *via* an adapter to a syringe filled with acetone and let the solvent flow until the inner cavity of the needle is completely free from residual deposits.

### Problem 5

Step 17: Yield is lower than expected.

### Potential solution

Vapourtec needles and annexed standard connecting tubes have an internal volume of approximately 700 μL. This volume must be considered when calculating the yield of the reaction, since it won’t be pumped in the reactor.

## Resource availability

### Lead contact

Further information and requests for resources and reagents should be directed to and will be fulfilled by the lead contact, Upendra Kumar Sharma (upendrakumar.sharma@kuleuven.be).

### Materials availability

All other data supporting the finding of this study are available within the article and the supplemental information, or from the lead contact upon reasonable request.

## Data Availability

• All data reported in this paper will be shared by the lead contact upon request. • This paper does not report original code. • Any additional information required to reanalyze the data reported in this paper is available from the lead contact upon request.
